# Safe Outpatient Dental Management of a Pediatric Patient With Mild Hemophilia A: A Case Report

**DOI:** 10.7759/cureus.97254

**Published:** 2025-11-19

**Authors:** Mireya Curiel-Ventura, Gabriela Torre-Delgadillo, Vanessa Alejandri-Gamboa, Ivette Pérez-Alfaro, Marlen Vitales-Noyola

**Affiliations:** 1 Postgraduate Program in Pediatric Dentistry, Faculty of Dentistry, Autonomous University of San Luis Potosi, San Luis Potosí, MEX; 2 Postgraduate Program in Pediatric Dentistry, Faculty of Dentistry, Autonomous University of San Luis Potosi, San Luis Potosi, MEX

**Keywords:** atraumatic restorative treatment, dental extraction, hemophilia-a, pediatric patient, pediatric preventive dentistry

## Abstract

Dental care in children with mild hemophilia A requires coordinated planning to prevent bleeding complications. An 11-year-old girl diagnosed recently with mild hemophilia A (factor VIII activity 46%) was referred for comprehensive dental care. Multidisciplinary planning with her pediatric hematologist was undertaken. One hour before multiple extractions (#55, #53, #65, #75 and a mesiodens), she received 1,000 IU of factor VIII. Extractions were completed using topical anesthesia for mobile primary teeth and limited lidocaine with epinephrine infiltration when needed, with local hemostasis achieved by gauze compression; no complications occurred. Subsequent restorative care included resin restorations (#21, #36, #12) and pit-and-fissure sealants (#46, #16, #26) during scheduled visits. The patient healed uneventfully and continued routine follow-up. In mild hemophilia A, individualized planning, pre-procedure factor replacement for invasive care, preference for infiltration techniques, and meticulous local hemostasis allow safe outpatient dental management.

## Introduction

Hemophilia A is an X-linked inherited bleeding disorder caused by factor VIII deficiency, characterized by impaired hemostasis and prolonged bleeding. Although females are typically carriers, some girls manifest the disease [1]. For dentists, the mouth is a high-risk field because routine procedures, extractions of mobile primary teeth, local anesthesia, scaling, impressions, and even trauma from erupting teeth or appliances can provoke mucosal bleeding that is difficult for families to control at home [2]. Safe care therefore depends on clear, pre-agreed protocols that match the planned dental procedure to the child’s baseline factor VIII activity and the need for replacement therapy or antifibrinolytics [3]. Dental decisions (e.g., favoring infiltration over mandibular blocks, staging invasive care in a single session after factor replacement, and using meticulous local hemostasis with pressure, sutures, or topical agents) directly influence outcomes [4].

Early integration of preventive dentistry is equally important. Caries control, sealants, minimally invasive restorations, and careful analgesic planning (avoiding nonsteroidal anti-inflammatory drugs (NSAIDs)) reduce the likelihood that the child will require future invasive procedures with higher bleeding risk [5]. This case illustrates a practical, interprofessional pathway that enabled comprehensive, complication-free dental treatment in a child with mild hemophilia A, emphasizing how tailored dental protocols are central to overall safety, not merely adjuncts to medical management.

## Case presentation

A 11-year-old girl from rural community was referred to the Pediatric Dentistry Clinic, Specialty of Pediatric Dentistry, Dentistry Faculty, previous hematologist had diagnosed symptomatic mild hemophilia A with 46% factor VIII activity and normal von Willebrand factor, the patient was under on-demand treatment, criteria for hemophilia A based on residual factor VIII (FVIII) activity, expressed as a percentage of normal plasma mild > 5% up to < 40%; moderate 1%-5%; severe < 1%. Family history included a father with moderate hemophilia A. The patient had previously undergone a dental extraction performed by a general dentist, during which she experienced excessive and uncontrolled bleeding. Following this episode, her parents brought her to the hospital, where she underwent hematologic evaluation and was subsequently diagnosed with mild hemophilia A.

**Figure 1:**
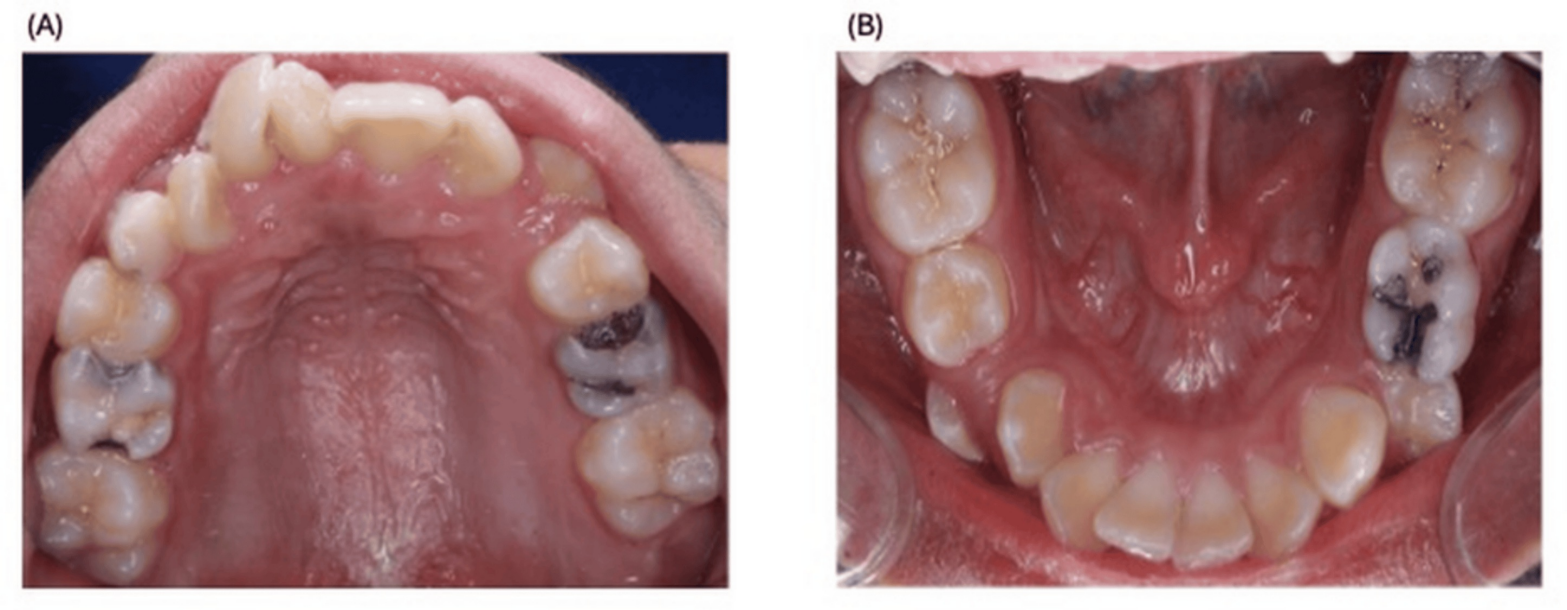
First-visit intraoral photographs: initial diagnostic assessment. A) Maxilla. B) Mandible.

At the first visit, a comprehensive assessment was performed. A complete medical and dental history was obtained. Extraoral and intraoral examinations were conducted, with detailed dental charting (odontogram), clinical photographs, and radiographs (Figure 1).

An integrated diagnosis and a phased treatment plan were established, prioritizing preventive measures and minimally invasive procedures. Given the history of mild hemophilia A, the plan was coordinated with Hematology, specifying factor VIII coverage for invasive procedures, a preference for infiltration anesthesia, meticulous local hemostasis, and analgesia avoiding NSAIDs. Ethics approval was granted by the Ethical and Research Committee of Stomatology Faculty, with the next code: CEI-FE-013-025 with approval date on April 3, 2025, and parents provided informed consent for treatment and publication. 

**Figure 2:**
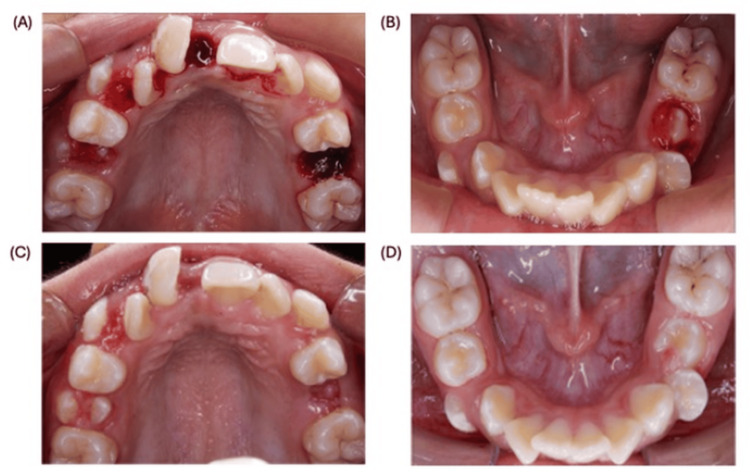
Postoperative photographic record showing recovery and procedural outcomes. A) 30 minutes postoperative, upper maxilla. B) 30 minutes postoperative, lower maxilla. C) One month postoperative, upper maxilla. D) One month postoperative, lower maxilla.

At the second visit, extractions were performed. To reduce repeated factor exposure, multiple extractions were scheduled for a single visit. 1,000 IU of factor VIII was administered one hour before the appointment. Extractions of next dental organs: #55, #53, #65, #75, and a mesiodens were performed. Mobile primary teeth were removed under 20% topical benzocaine gel (Zeyco, Zapopan, Mexico) alone; #65 and the mesiodens required limited infiltration with 2% lidocaine with epinephrine (1:100,000) (Zeyco). The decision to extract the severely mobile teeth rather than allowing them to exfoliate naturally is primarily to reduce the risk of bleeding. Since the patient is already receiving factor VIII coverage and requires extraction of the mesiodens and tooth #65, it is reasonable and efficient to perform the extractions of the mobile teeth during the same session. Hemostasis was obtained with sterile gauze; the patient was observed for 30 minutes and discharged with written instructions (including to avoid NSAIDs, use acetaminophen 500 mg for pain). No postoperative bleeding or complications occurred (Figure 2).

**Figure 3:**
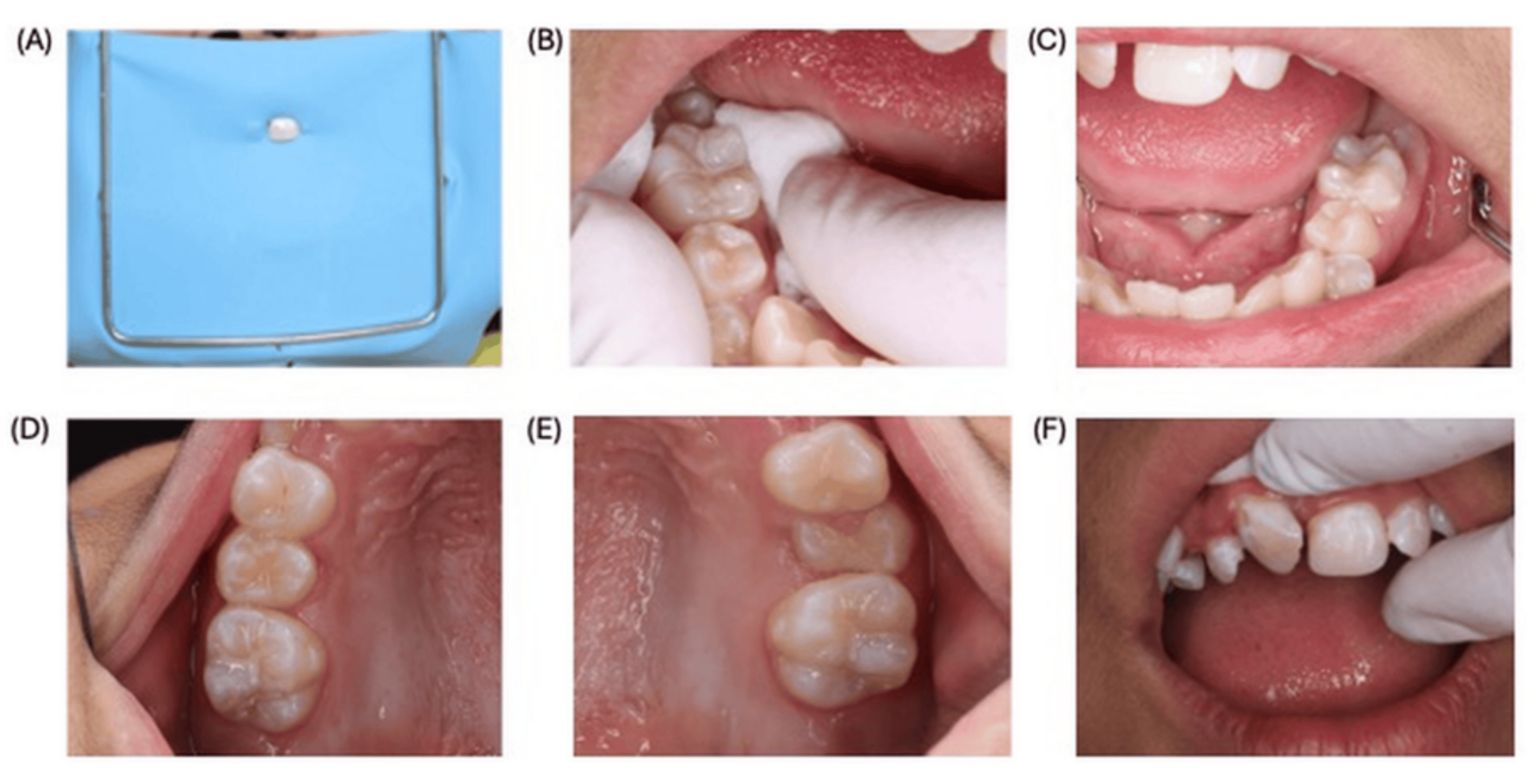
Photographic record corresponding to the rehabilitation of different teeth. A) Resin restoration, tooth #21. B) Sealant, tooth #46. C) Sealant, tooth #36. D) Sealant, tooth #16. E) Sealant, tooth #26. F) Resin restoration, tooth #12

Subsequent restorative and preventive care proceeded without factor coverage, as procedures were non-invasive and performed with careful isolation, resin restoration in #21 tooth, with rubber-dam isolation (young arch used, no clamp) and no local anesthesia, subsequent visit, pit-and-fissure sealant in #46 tooth and resin restoration in #36 tooth, using relative isolation (cotton rolls and high-volume suction, no dental clamp). Subsequent visit, sealants in #16 and #26 teeth, with relative isolation as above. In subsequent visit resin restoration in #12 tooth using partial isolation and an atraumatic restorative treatment (ART) approach; no dental clamp and using cotton-roll isolation (Figure 3). 

Additional visits addressed oral hygiene reinforcement, prophylaxis, and fluoride varnish because of suboptimal hygiene. The patient and her family received verbal and written oral hygiene instructions, which included proper tooth-brushing techniques and additional home-care measures such as flossing. Hematology recommended prophylactic factor VIII replacement secondary to menarche that coincidentally occurred, and encouraged follow-up. The patient was referred to orthodontics for malocclusion management. Throughout follow-up, there were no bleeding events related to dental care.

## Discussion

This case illustrates that comprehensive, office-based dental care can be delivered safely to a child with mild hemophilia A when management is protocol-driven and coordinated with coordinated with multidisciplinary healthcare team. Peri-operative planning centered on administering hemostatic factor VIII levels for invasive care, limiting cumulative factor exposure, and sequencing non-invasive procedures without replacement [6]. Short-interval pre-procedure factor VIII cover for multiple extractions in a single session balanced bleeding risk with practicality, reduced costs, logistics associated with repeated infusions, and avoided the incremental risk that accompanies multiple anesthetic events. Although adjuncts such as antifibrinolytics (e.g., tranexamic acid, topical or systemic) and local agents (gelatin sponge, fibrin sealants) are often recommended, meticulous mechanical hemostasis with gauze compression was sufficient in this patient when factor was optimized, underscoring that atraumatic technique can obviate additional measures in selected mild cases [6,7].

Anesthetic strategy is pivotal in these special cases [8]. We favored infiltration anesthesia and avoided mandibular nerve blocks, which carry a higher risk of deep-space hematoma and airway compromise in bleeding disorders. Infiltration for the maxilla and selected mandibular sites (especially primary teeth) provided adequate analgesia with a safer bleeding profile. For mobile primary teeth, topical anesthesia alone was adequate, further minimizing tissue trauma. The subsequent restorative phase emphasized minimally invasive dentistry, rubber-dam isolation without dental clamps when feasible, and relative isolation with cotton rolls and high-volume suction for sealants and conservative resin restorations. The atraumatic restorative treatment (ART) approach for #12 organ dental exemplifies a low-bleeding technique that preserves tooth structure and reduces the likelihood of pulp therapy or surgical interventions, which would demand more intensive hemostatic coverage.

Post-operative management also influences outcomes [9]. We used NSAID-sparing analgesia (acetaminophen) to avoid platelet function inhibition and provided explicit written home instructions with early follow-up, which are critical to detect rare, delayed bleeding. Importantly, dentistry does not occur in isolation for children with inherited bleeding disorders: preventive strategies such as caries control, fluoride varnish, sealants, oral-hygiene reinforcement, and dietary counseling directly reduce future indications for invasive procedures [10]. In our case, hygiene reinforcement and fluoride were incorporated into the care plan, and the patient was referred to orthodontics to address malocclusion in a planned, low-risk manner. Around menarche, on-demand factor was advised by Hematology to mitigate heavy menstrual bleeding, highlighting how broader developmental milestones intersect with dental safety.

From a risk-stratification standpoint, children with mild hemophilia A (factor VIII typically 5-40-50%) may be treated safely in ambulatory settings when procedures are triaged by invasiveness, factor levels are optimized for surgery, and teams are prepared with a bleeding action plan [11]. Nevertheless, not all lessons generalize, since this is a single mild-severity case without inhibitors and with normal von Willebrand factor; however, more complex scenarios (moderate/severe disease, inhibitor presence, extensive oral surgery) warrant distinct protocols, often incorporating antifibrinolytics and extended factor coverage. Finally, our experience reinforces two practical messages for dental teams: first, choose the lowest-trauma option that meets the therapeutic goal, as infiltration over blocks, ART and conservative resin over pulp therapy when appropriate, and cluster invasive care into a single, well-covered session whenever possible. The uneventful course, with no intra- or post-operative hemorrhage, supports that with targeted factor replacement, atraumatic technique, meticulous local hemostasis, and preventive emphasis, comprehensive outpatient dental treatment is both feasible and safe in children with mild hemophilia A.

## Conclusions

Comprehensive dental care in pediatric patients with mild hemophilia A can be safely delivered in an outpatient setting when management is individualized, coordinated with a multidisciplinary healthcare team, including hematology, and guided by appropriate preventive and surgical protocols. This includes pre-procedure factor replacement for invasive procedures, preference for infiltration over nerve blocks, meticulous local hemostasis, and continuous preventive maintenance to minimize the need for future invasive interventions.
